# Reply to Commentary to Skudlik et al. (2023): why a scoping review and why only Germany?

**DOI:** 10.1186/s12912-024-02078-6

**Published:** 2024-06-17

**Authors:** Stefanie Skudlik, Julian Hirt, Tobias Döringer, Regina Thalhammer, Katharina Lüftl, Martin Müller

**Affiliations:** 1 Centre for Research, Development and Technology Transfer, Rosenheim Technical University of Applied Sciences, Rosenheim, Germany; 2 Department for Primary Care and Health Services Research, Medical Faculty, Nursing Science and Interprofessional Care, Heidelberg University, Heidelberg, Germany; 3 Department of Health, Eastern Switzerland University of Applied Sciences, St. Gallen, Switzerland; 4 Department of Clinical Research, University Hospital Basel and University of Basel, Basel, Switzerland; 5 Faculty of Applied Health and Social Sciences, Rosenheim Technical University of Applied Sciences, Rosenheim, Germany

**Keywords:** Reply, Scoping review, Admission to nursing homes

## To the Editorial Board,

We have read the letter to the editor by Bich-Lien Nguyen entitled “Commentary to Skudlik et al. (2023): Why a scoping review and why only Germany?” with interest and reply briefly to address the concerns regarding methodological and content-wise decisions taken.

As outlined in our contribution, the presented scoping review [1] was part of a larger project to improve transitional care in the context of nursing home admission in Germany. There is some international evidence on transitional care interventions [2]. However, given the importance of contextualisation in the development of complex health and social care interventions [3–5], we aimed to appraise the existing research regarding German nursing home care. This is particularly relevant, as Germany's long-term care system (e.g. composition of personnel, resident populations, services) and education systems for nursing professionals largely differ from those in other countries [6–9]. As Sanford et al. (2015) [9] stated, research conducted in nursing homes in one country may not be directly applicable to nursing homes in another country, as the contexts can be very different, much like “comparing apples and oranges”. This was why we focused on Germany to inform the development of an intervention tailored to the German nursing home context.

Since a first orientating search revealed the absence of systematic and high-quality evidence, we decided to collect all potentially relevant studies irrespective of their type and quality to have a (a) comprehensive overview of the evidence and (b) to identify research gaps.

Following recent methodological guidance on scoping reviews [10, 11], it is a suitable and justified design to explore the evidence on a particular topic lead by a broad research question within a specific context (i.e., “what is know about challenges of nursing home admissions and care strategies in Germany?”) and to provide a systematic and comprehensive overview of the findings irrespective of the type of source (i.e., peer-reviewed reports and grey literature). Furthermore, the application of the outlined scoping review methodology helped us to directly inform the next steps of the development of a complex intervention which was the overarching goal of our approach [11].

We admit that the description of the nursing home setting in Germany could have been more detailed and take the opportunity to elaborate on it here: nursing homes in Germany vary in size, typically accommodating anywhere from a few dozen to several hundred residents. The population in nursing homes is usually mixed, comprising individuals with varying care needs [8, 12, 13]. Staff typically includes registered nurses, nursing aides, nursing assistants and a group of professionals known as “Betreuungsassistenten” or care assistants, which are trained to focus on the psychosocial aspects of care and social activities. Nursing homes in Germany are funded through a combination of public and private sources (statutory long-term care insurance system plus contribution towards the cost from residents / private funding). In general, nursing homes in Germany do not provide post-acute rehabilitation [12, 13]. There are efforts to strengthen end-of-life care within German nursing homes, however, many residents in the final phase are still being transferred to hospitals [14, 15].

We hope that our elaboration provided additional insights into the conceptualisation and context of our review which we experienced as fundamental for our project.

In summary, we strongly believe that research designs, no matter whether original or secondary research should be determined by the research question rather than a generic hierarchy. As such, our very focussed scoping review was the right decision to summarise the country-specific published knowledge which we needed to further develop our project.

## Data Availability

No datasets were generated or analysed during the current study.

## References

[1] Skudlik S, Hirt J, Döringer T, Thalhammer R, Lüftl K, Prodinger B, Müller M (2023). Challenges and care strategies associated with the admission to nursing homes in Germany: a scoping review. BMC Nurs.

[2] Groenvynck L, Fakha A, de Boer B, Hamers JPH, van Achterberg T, van Rossum E, Verbeek H (2022). Interventions to Improve the Transition From Home to a Nursing Home: A Scoping Review. Gerontologist.

[3] Moore GF, Audrey S, Barker M, Bond L, Bonell C, Hardeman W (2015). Process evaluation of complex interventions: Medical Research Council guidance. BMJ.

[4] Grant A, Treweek S, Dreischulte T, Foy R, Guthrie B (2013). Process evaluations for cluster-randomised trials of complex interventions: a proposed framework for design and reporting. Trials.

[5] Skivington K, Matthews L, Simpson SA, Craig P, Baird J, Blazeby JM (2021). A new framework for developing and evaluating complex interventions: update of Medical Research Council guidance. BMJ.

[6] Ewers  M, Lehmann  Y, Jacobs  K, Kuhlmey  A, Greß  S, Klauber  J, Schwinger  A (2020). Hochschulisch qualifizierte Pflegende in der Langzeitversorgung?!. Pflege-Report 2019.

[7] Meng M, Peters M, Dorin L (2022). Erste Sondererhebung des BIBB-Pflegepanels: ein aktueller Überblick zu berufsqualifizierenden Pflegestudiengängen.

[8] Kraus M, Fößleitner S, Riedel  M, Jacobs  K, Kuhlmey  A, Greß  S, Klauber  J, Schwinger  A (2020). Pflegesysteme im internationalen Vergleich. Neuausrichtung von Versorgung und Finanzierung.

[9] Sanford AM, Orrell M, Tolson D, Abbatecola AM, Arai H, Bauer JM (2015). An International Definition for “Nursing Home”. J Am Med Dir Assoc.

[10] Peters MDJ, Marnie C, Tricco AC, Pollock D, Munn Z, Alexander L (2021). Updated methodological guidance for the conduct of scoping reviews. JBI Evid Implement.

[11] Munn Z, Pollock D, Khalil H, Alexander L, Mclnerney P, Godfrey CM (2022). What are scoping reviews? Providing a formal definition of scoping reviews as a type of evidence synthesis. JBI Evid Synth.

[12] Blümel M, Spranger A, Achstetter K, Maresso A, Busse R (2020). Germany: Health System Review. Health Syst Transit.

[13] Bundesministerium für Gesundheit. Siebter Pflegebericht: Bericht der Bundesregierung über die Entwicklung der Pflegeversicherung und den Stand der pflegerischen Versorgung in der Bundesrepublik Deutschland Berichtszeitraum: 2016–2019. 2021. https://www.bundesgesundheitsministerium.de/fileadmin/Dateien/3_Downloads/P/Pflegebericht/Siebter_Pflegebericht_barrierefrei.pdf. Accessed 6 Mar 2023.

[14] Allers K, Fassmer AM, Spreckelsen O, Hoffmann F (2020). End-of-life care of nursing home residents: A survey among general practitioners in northwestern Germany. Geriatr Gerontol Int.

[15] Strautmann A, Allers K, Fassmer AM, Hoffmann F (2020). Nursing home staff's perspective on end-of-life care of German nursing home residents: a cross-sectional survey. BMC Palliat Care.

